# A review of the correlation of tergites, sternites, and leg pairs in diplopods

**DOI:** 10.1186/1742-9994-3-2

**Published:** 2006-02-02

**Authors:** Ralf Janssen, Nikola-Michael Prpic, Wim GM Damen

**Affiliations:** 1 Department for Evolutionary Genetics, Institute for Genetics, University of Cologne, Zülpicher Straße 47, 50674 Köln, Germany

## Abstract

In some arthropods there is a discrepancy in the number of dorsal tergites compared to the number of ventral sternites and leg pairs. The posterior tergites of the Diplopoda (millipedes) each cover two sternites and two pairs of legs. This segment arrangement is called diplosegmentation. The molecular nature of diplosegmentation is still unknown. There are even conflicting theories on the way the tergites and sternites/leg pairs should be correlated to each other. The different theories are based either on embryological analyses or on studies of the adult morphology and turned out to be not compatible with each other. We have previously used the expression patterns of segmentation genes in the pill millipede *Glomeris marginata *(Myriapoda: Diplopoda) to study millipede segmentation. Here we review the existing models on the alignment of tergites and leg pairs in millipedes with special emphasis on the implications the gene expression data have on the debate of tergite and leg pair assignment in millipedes. The remarkable outcome of the gene expression analysis was that (1) there is no coupling of dorsal and ventral segmentation and, importantly, that (2) the boundaries delimiting the tergites do neither correlate to the embryonic boundaries of the dorsal embryonic segments nor to the boundaries of the ventral embryonic segments. Using these new insights, we critically reinvestigated the correlation of tergites, sternites, and leg pairs in millipedes. Our model, which takes into account that the tergite boundaries are different from the dorsal embryonic segment boundaries, provides a solution of the problem of tergite to sternite/leg pair correlation in basal milipedes with non-fused exoskeletal elements and also has implications for derived species with exoskeletal rings. Moreover, lack of coupling of dorsal and ventral segmentation may also explain the discrepancy in numbers of dorsal tergites and ventral leg pairs seen in some other arthropods.

## Introduction

**Figure 1:**
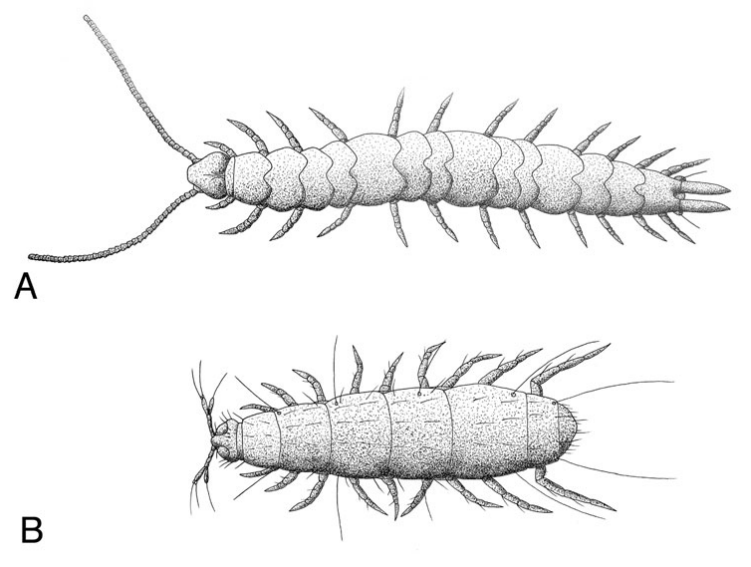
Myriapod groups with differences in dorsal and ventral segmentation. (A) A male *Scutigerella immaculata *(Myriapoda: Symphyla). Note the different number of dorsal units (15 tergites) and ventral units (twelve pairs of trunk legs). (B) Specimen of *Pauropus huxleyi *(Myriapoda: Pauropoda). Note that nine pairs of trunk legs are present, contrasting with only seven tergites (including anal plate). Both species are shown in dorsal aspect, anterior to the left. After [19].

If one studies insects, one is familiar with the fact that the number of metameric units counted on the dorsal side of the body usually matches the number on the ventral side. For example, the insect thorax has three tergites on the dorsal side corresponding to three sternites and three pairs of legs on the ventral side. Such a match between the number of dorsal (tergites) and ventral structures (sternites, leg pairs) is seen in most arthropods. There are, however, some arthropod groups that are more variable in terms of dorsal versus ventral metameric units. Symphyla, for example, which are a small group of myriapods (about 150 species worldwide) (Fig. 1A). When counted on the dorsal side, the trunk of most symphylan species has 15 tergites, which suggests the presence of 15 trunk segments. However, if one counts ventral elements in the trunk, for example the leg pairs, one arrives at a count of only twelve! The opposite phenomenon is seen e.g. in the Pauropoda, another group of myriapods (Fig. 1B). In most pauropods the dorsal count of trunk tergites is seven (including the anal plate). The number of leg pairs on the ventral side of the trunk, however, is higher, mostly nine. The phenomenon of an unequal number of ventral and dorsal metameric units is not restricted to these particular groups, but is also seen in other arthropod groups like e.g. the Diplopoda (millipedes), and some crustaceans, like the Notostraca. Clearly, in these species the number of segmental units is not identical on the ventral and the dorsal side, raising the question how this can be explained.

**Figure 2:**
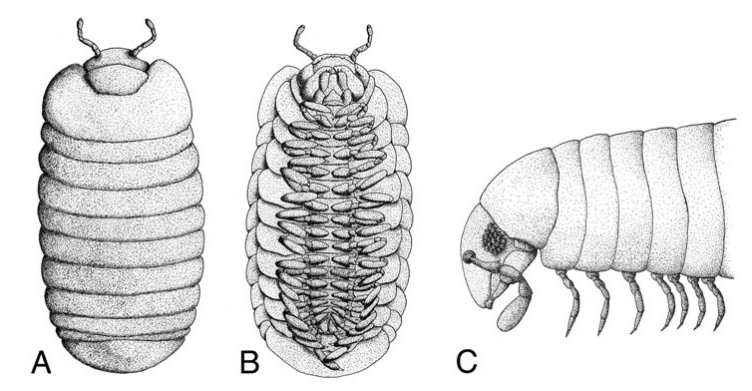
Dorsoventral discrepancies in millipedes (Myriapoda: Diplopoda). (A) Dorsal view of a female *Glomeris marginata*, anterior to the top. This species has twelve tergites. (B) Ventral view of a female *Glomeris marginata*. This view reveals the presence of 17 pairs of trunk legs. Also note the lateral pleurites, which are not fused to the tergites or sternites. This species does not form exoskeletal rings. (C) A generalized juliform diplopod, lateral aspect, anterior to the left. In this diplopod group sternites, pleurites and tergites are fused into rigid armour rings, which give the trunk its annulated ("segmented") appearance. Only the first tergite, the neck-shield or collum, is free, *i.e. *not fused to any other component. Note that the first three armour rings bear a single pair of legs, whereas all following rings have two leg-pairs each. A and B are drawn after specimens preserved in methanol. C has been modified and simplified from [19].

We try to tackle this problem using as a model the pill millipede *Glomeris marginata *(Myriapoda, Diplopoda). When viewed from the dorsal side, the trunk of this species has twelve tergites (Fig. 2A). The first tergite is relatively small and has a specific name, the so-called collum. The next tergite, tergite II, is the largest, and is followed by nine tergites (tergite III–XI) of roughly the same size. The last tergite, tergite XII, has the form of a shield. This composition thus suggests that the trunk of *Glomeris *consists of twelve segments. If the animal is turned over on its back, however, the number of leg pairs suggests a higher number of trunk segments: female *Glomeris *have 17 pairs of trunk legs (Fig. 2B), and males even have 19 pairs of legs. The question is now: How can the 17/19 pairs of legs be correlated with the 12 tergites? A look to the ventral side of a *Glomeris *specimen (Fig. 2B) demonstrates that it is virtually impossible to perform this correlation task: there is no obvious link between the tergites on the dorsal side and the sternites and leg pairs on the ventral side.

## A model for tergite-sternite-leg pair assignment based on adult ring-forming millipedes

Fortunately, however, there are other millipede species, e.g. the members of Juliformia (Fig. 2C) that have their tergites and sternites fused together into rigid armour rings (for an overview see [1,2]). Each pair of legs is jointed to a sternite that is fused to a tergite via the lateral pleurites, in this way making leg pair/tergite alignment seemingly straightforward and unambiguous. In these animals, the first tergite (collum) is free (i.e. not fused to any ventral component). The second tergite is fused with the sternites of the first pair of legs into the first cuticular ring (ring 1) (see Fig. 2C). The third and fourth tergites are fused with the second and third trunk sternites, respectively, thus forming ring 2 and 3. The anterior part of the juliform trunk, therefore, consists of a free collum, followed by three rigid rings, each of which is bearing a single pair of legs. Posterior to this something surprising happens: each tergite is fused not to a single sternite, but to a pair of sternites. The result: starting with ring 4 each ring has two pairs of legs (see Fig. 2C).

**Figure 3:**
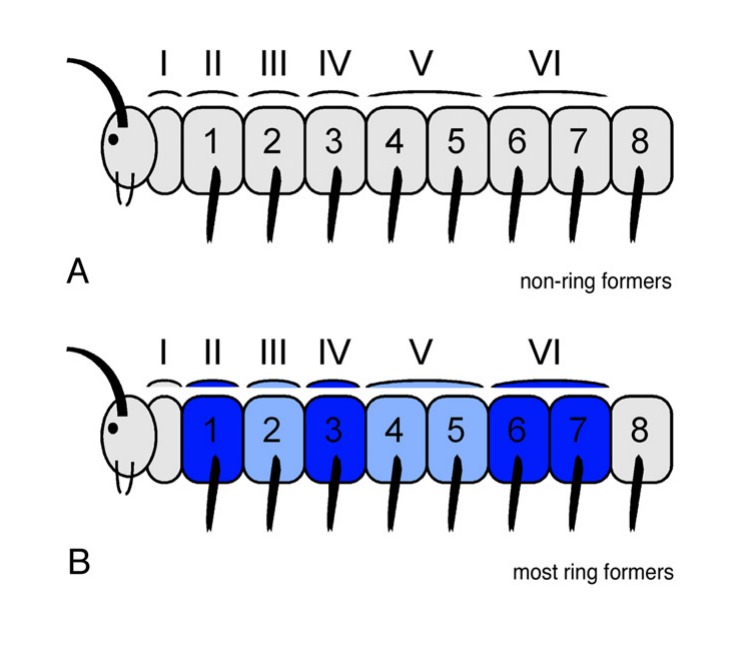
Model for tergite – sternite/leg pair correlation in millipedes based on morphological evidence from adult ring-forming millipedes. The model is based on the way the tergites and sternites are fused into rings in ring-forming millipede species. Tergites are denoted with roman numbers (I–VI), sternites and leg pairs are denoted with Arabic numbers (1–8). For explanatory reasons, the post-maxillary segment is drawn between the head and the first trunk segment; in fact this segment is the last segment of the head. The way the tergites and sternites fuse into rings in ring-forming species as indicated by same shade of blue for one ring in panel B. This fusion into rings is used in this model to correlate tergites with sternites/leg pairs. The hypothetical extrapolation for the tergite – sternite/leg pair correlation in non-ring-forming species is depicted in panel A. For details, see text.

In this way, the correlation of leg pairs and tergites leads to the outcome as summarized in Fig. 3B: tergite II correlates with trunk leg pair 1; tergite I (the collum), therefore, cannot correlate with any leg pair and is attributed to the last head segment, the post-maxillary segment, that in millipedes does not have appendages (dating back to [3]). Tergite III and IV correlate with leg pair 2 and 3, respectively. Tergite V and all following tergites correlate with two leg pairs each: tergite V correlates with leg pairs 4 and 5, tergite VI with leg pairs 6 and 7, and so forth.

In order to distinguish between the tergites that correlate with only one leg pair and those that correlate with two leg pairs, tergites II, III, and IV are called haplotergites, whereas all following tergites are so-called diplotergites. The phenomenon that one dorsal segmental unit (one diplotergite) is correlating with two ventral segmental units (two sternites and two leg pairs) is called diplosegmentation.

As already mentioned above, in non-ring-forming species with free tergites, pleurites and sternites, the correlation of leg pairs with tergites is unclear since they are not fused into rings. However, the results from ring-forming species can be extrapolated to non-ring-forming species, resulting in a similar assignment (Fig. 3A). Thus, according to the correlation scheme derived from the study of ring-forming millipedes, the millipede trunk has three "haplosegments" followed by a number of "diplosegments" (Fig. 3).

## Another model for tergite-sternite-leg pair assignment based on embryology in non-ring-forming millipedes

**Figure 4:**
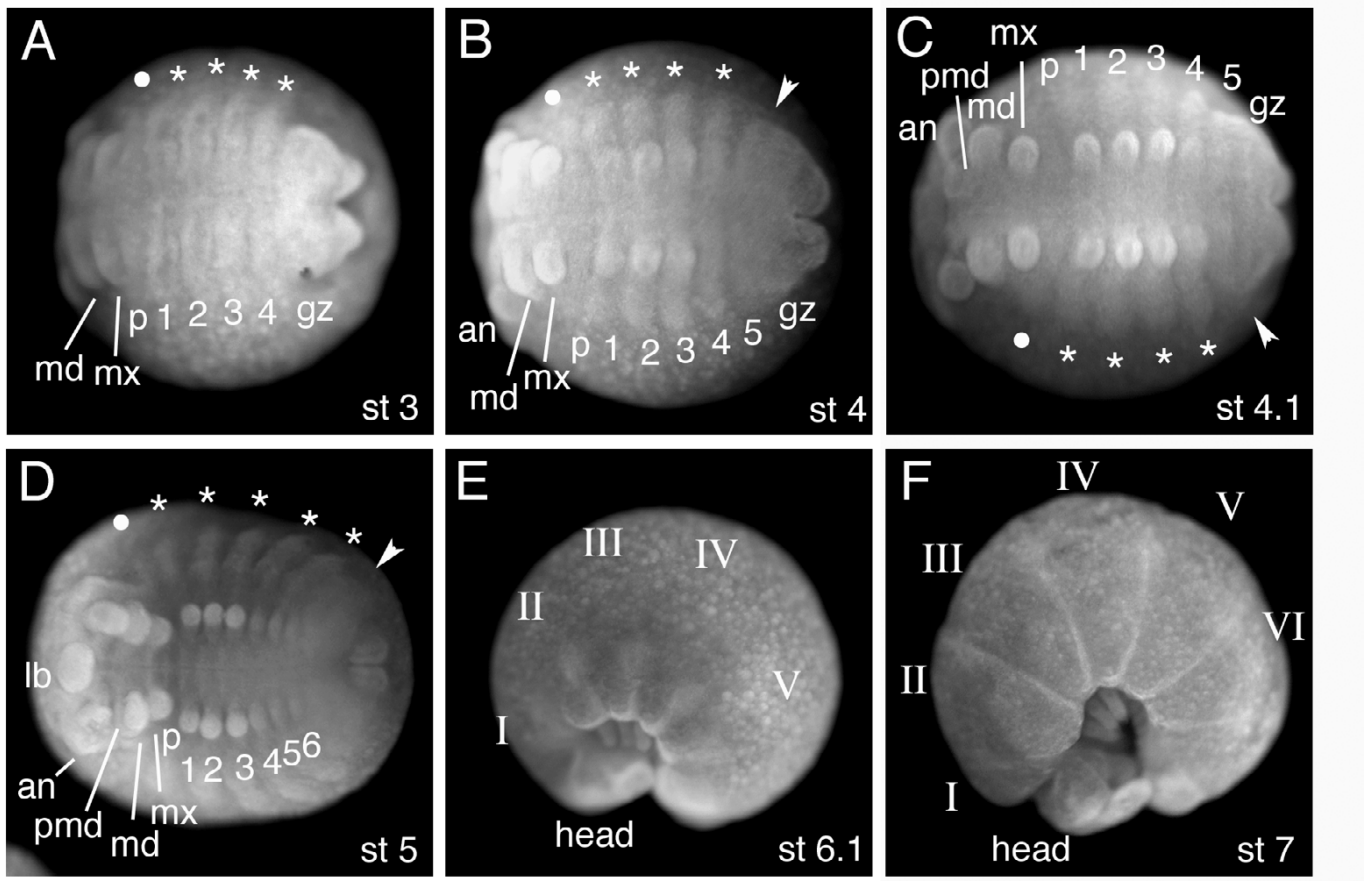
Summary of the formation of dorsal tissue during embryonic development in *Glomeris marginata*. Embryos are stained with the nuclear fluorescent dye DAPI. The developmental stages are as indicated; note that these stages are before dorsal closure, the lateral tissue on both sides of the germ band is the future dorsal tissue (details see text). (A) Trunk region of a stage 3 embryo (staging see [4,6]). At this stage the head segments (only gnathal segments visible here) and the first four metameric units of the ventral trunk have formed. Additional trunk metameric units will be added from the posterior growth zone (gz). At this stage the *de novo *formation and outgrowth of the dorsal tissue (lateral plates) commences (filled circle and asterisks). The asterisks mark the dorsal tissue aligned with the trunk segments. The filled circle marks the common dorsal tissue of the gnathal segments. (B) Stage 4 embryo. The outgrowth of the lateral plates proceeds (asterisks). Note that the growth zone now gives rise to ventral as well as dorsal tissue (arrowhead). (C) Stage 4.1 embryo. The fifth ventral trunk metamere has formed from the growth zone. (D) Stage 5 embryo. The embryo starts to bend in to form the characteristic shape of a "pill". The sixth ventral trunk metamere has formed. Note that the fifth and sixth ventral metameres share one common dorsal tissue that has been added from the growth zone. (E) Stage 6.1 embryo. The embryo has bent in completely. Roman numerals denote the future tergites. (F) Stage 7 embryo. The bipartite body plan of the millipede composed of head and trunk becomes clear. The outgrowth of the dorsal plates has almost finished and the dorsal tissues meet at the back of the embryo to complete dorsal closure. Note that the constrictions between the tergites are clearly visible at this late embryonic stage. Anterior is always to the left. A–D are ventral views, E and F are lateral views. Abbreviations: lb: labrum; an: antennal segment; pmd: pre-mandibular segment; md: mandibular segment; mx: maxillary segment; p: post-maxillary segment; gz: growth zone; 1–6: ventral leg bearing metameric unit 1–6; I–VI: tergites I–VI.

**Figure 5:**
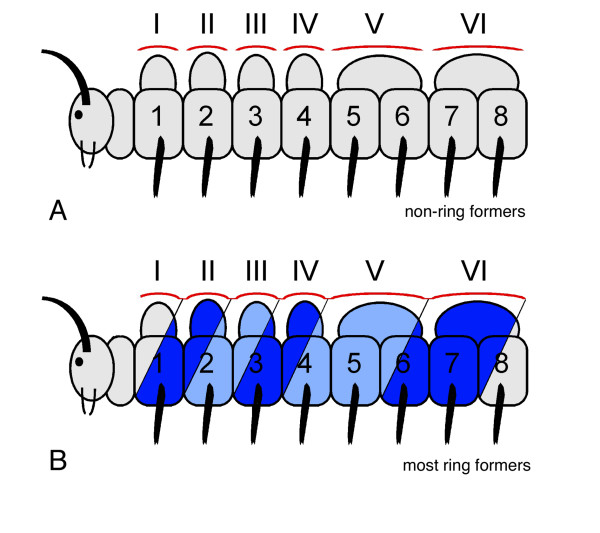
Model for tergite – sternite/leg pair correlation in millipedes based on embryonic evidence from basal non-ring-forming millipedes. The model is based on the way the dorsal and ventral metameric units form in the embryo. Tergites are denoted with roman numbers (I–VI), sternites and leg pairs are denoted with Arabic numbers (1–8). This model assumes that the tergites are directly derived from the dorsal embryonic segments, and accordingly correlates the tergites with the sternites and leg pairs (panel A). Panel B: hypothetical extrapolated model for ring-forming millipedes. The situation in ring-forming millipedes can only be explained by assuming a shift in the fusion of dorsal and ventral exoskeletal elements (indicated as blue shades in panel B). For instance, the first ring in the ring-forming millipedes forms via the fusion of tergite II to the leg pair 1, the second ring via the fusion of tergite III with leg pair 2, and so on; the different shades of blue in panel B represent the rings in adult specimen. The first tergite is the collum that is not fused to any leg pair. This model leads to a different tergite – sternite/leg pair correlation as in the 'adult morphological' model in Fig. 3. For details, see text.

Embryological studies in non-ring-forming species, however, do not support the extrapolation as depicted in Figure 3 and suggest another assignment. Dohle [4] studied the embryogenesis of *Glomeris marginata *and was able to show that each leg pair in *Glomeris *grows from a metameric unit in a way similar to the other arthropods. He found no evidence for single embryonic metameric units bearing two leg pairs each; rather also between the leg pairs of the alleged diplosegments (e.g. between trunk leg pair 4 and 5) there are distinct boundaries dividing the ventral portion of the germ band into units bearing a single pair of legs throughout. However, Dohle [4] discovered that the developing germ band grows extensions towards the dorsal side that he termed "Seitenplatten" (lateral plates) (Fig 4A–D). The first four metameric units of the trunk each develop a single lateral plate on each side, but the following metameric units form lateral plates pair wise. Given that the lateral plates, despite their name, will grow towards the dorsal side and form the dorsal epidermis, Dohle [4] assumed that the lateral plates give rise to the tergites on the dorsal side (Fig 4E,F). The resulting model for alignment of leg pairs and tergites is shown in Fig. 5A: The first four metameric units in the trunk develop single lateral plates each, that after dorsal closure segregate a single tergite each. The metameric units 5 and 6 in the trunk and all following pairs develop lateral plates jointly, so that after the segregation of cuticle a single tergite is formed covering two ventral metameric units each. Each of the ventral metameric units bears one pair of legs. Dohle [4] concluded that the first four leg bearing units are "haplosegments", and that the following units that develop joint lateral plates in a pair-wise manner are "diplosegments". This model of tergite/leg pair alignment very obviously differs from the previous model (compare Fig. 3A with Fig. 5A). In addition, the tergite/leg pair correlation that Dohle [4] postulated for non-ring-forming species clearly contradicts the actual correlations as they are found in ring-forming species (compare Fig. 5B with Fig. 3B). One possible explanation for these contradictory results is, that the tergite/leg pair correlation is different in non-ring-forming and ring-forming species. However, this would be very unlikely and Dohle [4,5] offers a different explanation (Fig. 5B): He assumes that in ring-forming species tergites and sternites fuse in a shifted manner (e.g. tergite II (that in his model belongs to leg-bearing unit 2) fuses with the sternite of leg-bearing unit 1 instead of the sternite of leg bearing unit 2 directly opposite).

Both models most significantly differ in the assignment and location of the tergites and the diplosegments, both of which are metameric structures. We decided to study the long-standing issue of the discrepancy in assignment of dorsal and ventral metameric units by analyzing genes controlling segmentation in the millipede *Glomeris marginata *[6]. Our results show surprising differences in expression of these genes in dorsal and ventral metameric units. In our previous paper [6] we focused on the different mechanisms that must act during the formation of the dorsal and ventral metameric units in the embryo. In the present paper we discuss in detail the implications the lack of coupling of dorsal and ventral segmentation has for the debate on the correlation of tergites with sternites and leg pairs. We review here the evidence for an alternative model for tergite – sternite/leg pair assignment that provides a solution for the problems in the previous models.

## Segmentation genes and segment formation in arthropods

Segmentation genes have been studied in great detail in the fruit fly *Drosophila melanogaster*, and as a consequence, *Drosophila *segmentation still forms a paradigm for segmentation. The Nobel Prize awarded *D. melanogaster *mutagenesis screen reported by Nüsslein-Volhard and Wieschaus [7] revealed several classes of mutant segmentation phenotypes. The affected genes in these mutants are the so-called segmentation genes and were shown to act in a hierarchic gene cascade to divide the early embryo into a repeating series of segmental units along the anterior posterior axis. One of the classes of segmentation genes are the segment-polarity genes that are responsible for establishing and maintaining the boundaries of the initial metameric units, the parasegments. These genes also define the A-P polarity within each metameric unit.

The best-studied segment-polarity gene is the *engrailed *gene. The *engrailed *gene is expressed in segmentally reiterated stripes in the embryo. In *Drosophila*, another segment-polarity gene, the *wingless *gene, is active in cells just anteriorly adjacent to the stripe of *engrailed *expressing cells. The *engrailed *and *wingless *expressing cells are mutually exclusive and define an important boundary between them, the parasegmental boundary. Genetic and molecular studies have shown that the parasegments are fundamental units in the design of the early *Drosophila *embryo [8-11].

The analysis of *Drosophila *segment-polarity gene homologs in other arthropods, like more primitive insects, crustaceans, centipedes, and spiders, revealed that the role of these genes in defining the parasegmental boundary represents a conserved function among arthropods (e.g. [12,13]). Also in these arthropods *wingless*/*Wnt *genes are active in cells anteriorly adjacent to the *engrailed *expressing cells. Detailed analyses, *e.g. *in the spider *Cupiennius salei *and the centipede *Lithobius atkinsoni*, demonstrated that the border between the *engrailed *and *wingless/Wnt *expressing cells also defines the important boundary of the parasegment, similar as in insects [12,13]. Also in crustaceans *engrailed *expression is associated with units comparable to parasegments [14]. The expression of these genes thus forms an excellent marker for the formation of the segmental units in arthropods.

## Segmentation genes in dorsal and ventral embryonic segments of the millipede *Glomeris*

**Figure 6:**
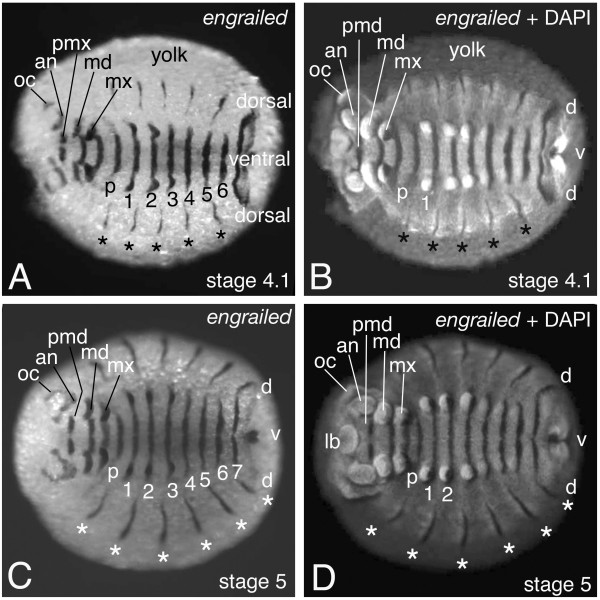
Expression of the *engrailed *gene in the embryo of the millipede *Glomeris marginata*. Shown is the localisation of *engrailed *transcripts via in situ hybridisation. The embryo proper is located on the surface of a large yolk mass that is consumed as the embryo develops. In the stages shown, the yolk mass is still very large, so that the embryo proper is still open dorsally. This is the reason why the future dorsal tissue is still located "laterally", i.e. next to the ventral tissue (indicated in the figures). This will change in very late stages, when the yolk is consumed entirely and the "lateral" portions of the germ band fuse dorsally (dorsal closure). The ventral *engrailed *expression marks the posterior part of the ventral segmental units. The ventral stripes are marked with p,1,2,3,4,5,6,7. Dorsally, the *engrailed *transcripts are in stripes (asterisks) localised at a different intrasegmental position as on the ventral side. The dorsal stripe of *engrailed *expression is not associated with any morphological structure at this stage, but is in the middle of the dorsal metameric units, which also becomes obvious if one compares with figure 7 B and D. The location of the future ventral and dorsal tissue is marked (dorsal-ventral-dorsal or d-v-d). Note that the dorsal tissue is on the lateral part on both sides of the germ band at this stage; during dorsal closure these tissues fuse on the dorsal side to give rise to one dorsal tissue. The head segments are marked in the embryo in panel D, for details we refer to [4,6]. (A and B): embryo at stage 4.1, (C and D): embryo at stage 5 (staging see [4,6]). Panel A and C show the bright field micrograph of the in situ hybridisation staining; panel B and D show the epifluorescence image of the embryo in A and C, respectively, in which the nuclear DAPI staining becomes bright, and the in situ hybridisation staining appears dark due to quenching of the fluorescence. Abbreviations: d: dorsal; v: ventral; lb: labrum; oc: ocular segment; an: antennal segment; pmd: pre-mandibular segment; md: mandibular segment; mx: maxillary segment; p: post-maxillary segment; 1–7: ventral leg bearing segment 1–7.

**Figure 7:**
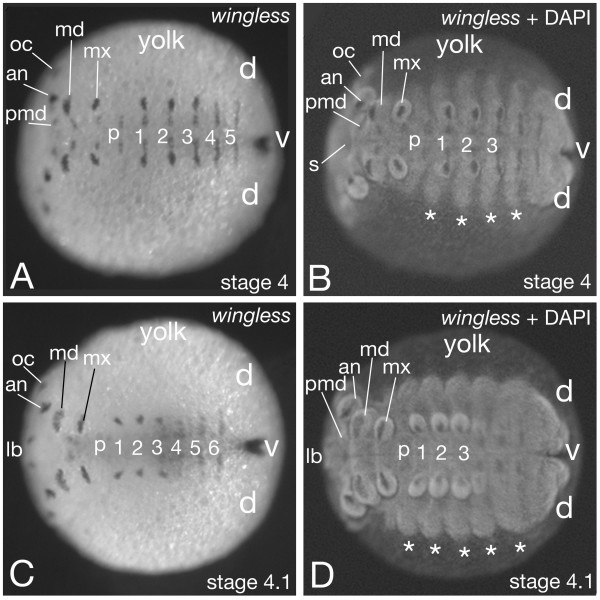
Expression of the *wingless *gene in the embryo of the millipede *Glomeris marginata*. Shown is the localisation of *wingless *transcripts via in situ hybridisation. The ventral *wingless *expression is located just anterior to the position of the *engrailed *stripe (see Fig 6). Dorsally, no *wingless *transcripts are detected. The asterisks mark the dorsal metameric units, which are the lateral plates of Dohle [4]. The location of the future ventral (v) and dorsal (d) tissue is marked, see also figure 6. (A and B): embryo at stage 4, (C and D): embryo at stage 4.1 (staging see [4,6]). Panel A and C show the bright field micrograph of the in situ hybridisation staining; panel B and D show the epifluorescence image of the embryo in A and C, respectively, in which the nuclear DAPI staining becomes bright, and the in situ hybridisation staining appears dark due to quenching of the fluorescence. Abbreviations: d: dorsal; v: ventral; lb: labrum; an: antennal segment; pmd: pre-mandibular segment; md: mandibular segment; mx: maxillary segment; p: post-maxillary segment; 1–6: ventral leg bearing segment 1–6, s: stomadeum.

As the role of these segment-polarity genes appears to be conserved among arthropods, we decided to analyze segment-polarity genes in the diplopod *Glomeris marginata *[6]. We discovered a remarkable difference in the expression of these genes between the ventral and the dorsal side of the embryo (Figs. 6 &7). Ventrally, the expression is identical to what is seen in other arthropods: *engrailed *is expressed in segmentally reiterated stripes (Fig. 6) and *wingless *is expressed in stripes that are at a position just anterior to the *engrailed *stripes (Fig. 7). The boundaries between the ventral metameric units are directly behind each *engrailed *stripe. This is identical to the segments of other arthropods where *engrailed *is also expressed in the posterior part of the segments. We therefore designate the ventral metameric units as: ventral embryonic segments. The expression of *engrailed *and *wingless *suggests that the segment-polarity gene network as present in all arthropods analyzed so far also acts in the ventral embryonic segments in *Glomeris*. The expression of additional genes like *cubitus interruptus *and *hedgehog *[6] supports this notion.

On the dorsal side the gene expression patterns are significantly different. As described above, development of the presumptive dorsal tissue in *Glomeris *starts later than the ventral tissue, and is forming the so-called lateral plates [4,6] (Fig 4). The anterior portion of the germ band up to the 4th ventral embryonic segment of the trunk initially comprises ventral tissue only. At stage 3 the development of the dorsal tissue commences (Fig 4AB) and *engrailed *expression is activated *de novo *in this tissue (Fig. 6A,B asterisks). These dorsal *engrailed *stripes, however, have no connection with the ventral *engrailed *stripes and in contrast to the ventral *engrailed *stripes are not associated with the morphologically visible grooves. Furthermore, the dorsal *engrailed *stripes are not aligned with the ventral *engrailed *stripes, but are shifted in relation to them. More posterior the dorsal tissue forms simultaneously with the ventral tissue. Here, the *engrailed *gene is expressed in each ventral embryonic segment, while the gene is only expressed in the dorsal tissue corresponding to every other ventral embryonic segment [6]. On the dorsal side there are thus reiterated stripes of *engrailed *expression (Fig. 6), but they do not correspond in a one-to-one fashion to the *engrailed *stripes on the ventral side, they are not associated with a morphological boundary, and, surprisingly, they are not associated with *wingless *expression (Fig. 7) (see also [6]). The lack of *wingless *expression in the dorsal tissue implies that there cannot be an interface of *engrailed *and *wingless *expressing cells to define a boundary, like on the ventral side. This argument is strengthened by the lack of *cubitus interruptus *gene expression anterior to the dorsal stripes of *engrailed *expression [6]. Cubitus interruptus is the activator of *wingless *gene expression. These data imply that the genetic interactions must be different in the dorsal and ventral tissue of *Glomeris*. In order to acknowledge this fact we regard the dorsal metameric units, the lateral plates [4], as segmental units and in order to distinguish them from the ventral embryonic segments we designate them as: dorsal embryonic segments

There thus appears to be a lack of coupling of dorsal and ventral segmentation as is obvious from several pieces of evidence. (1) The anterior dorsal tissue forms later than the ventral one and (2) the *engrailed *stripes appear *de novo *in this anterior dorsal tissue. (3) The dorsal *engrailed *stripes are not located near the morphological boundaries between the metameric units and (4) the dorsal metameric units differ in number from the ventral ones. (5) But most significantly, the genetic interactions must be different as *wingless *expression is missing in dorsal tissue. Dorsal and ventral segmentation thus are not coupled in *Glomeris*.

## Dorsal *engrailed *expression coincides with tergite borders

**Figure 8:**
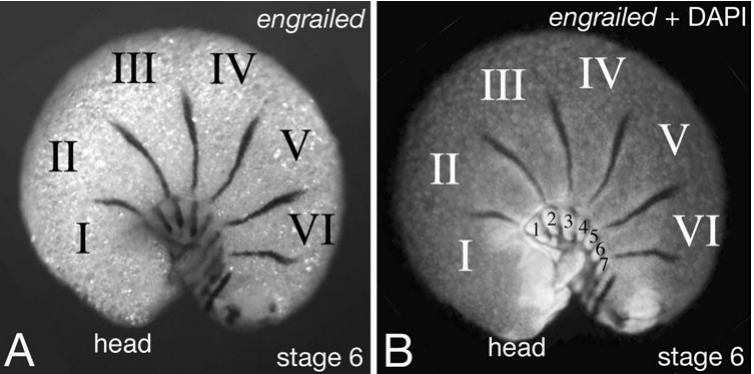
Dorsal *engrailed *stripes mark the forming tergite borders. Shown is an in situ hybridisation for *engrailed *in a stage 6 *Glomeris *embryo. At this stage the embryo rolls in (see leg pairs at ventral side in B) and it becomes clear that the dorsal stripes of *engrailed *expression are co-localised with the tergite borders. Panel A shows a bright field image of the in situ hybridisation staining, panel B shows an epifluorescence image of the same embryo, in which the nuclear DAPI staining becomes bright, and the in situ hybridisation staining appears dark due to quenching of the fluorescence. The future tergites are denoted with roman numbers (I–VI).

The ventral *engrailed *expression is associated with *wingless *expression and thus with the parasegmental boundary like in all other arthropods studied so far. By contrast, the dorsal *engrailed *expression in early embryonic stages is not associated with any morphologically visible boundary. There are conspicuous constrictions between the dorsal embryonic segments (termed "lateral plates" [4]), but the dorsal *engrailed *expression is in cells approximately in the middle of such a dorsal embryonic segment and not near the boundaries of them (Fig. 6). However, at later stages of development secondary constrictions form at the location of the dorsal *engrailed *stripes and in these older embryos it can be seen that these constrictions are the boundaries between the tergites (Fig. 4 and Fig. 8). The expression of *engrailed *on the dorsal side of *Glomeris *thus prefigures the location of the tergite boundaries, and it is likely that this gene plays a role in defining and establishing this developmental boundary.

## Sternite – tergite correlation in diplopods

**Figure 9:**
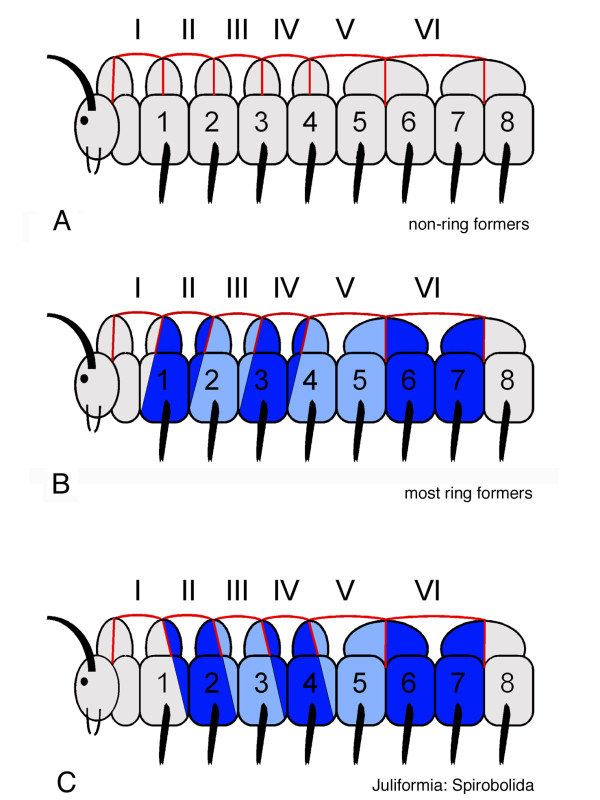
Model for tergite – sternite/leg pair correlation in millipedes based on the lack of coupling of dorsal and ventral segmentation. Our model is based on the way the dorsal and ventral metameric units form in the embryo as well as on gene expression data that imply a lack of coupling of dorsal and ventral segmentation. Key in the model is the fact that the tergite boundaries do neither correlate to dorsal embryonic segment boundaries, nor to ventral embryonic segment boundaries. Tergites are denoted with roman numbers (I–VI), sternites and leg pairs are denoted with Arabic numbers (1–8). (A) The tergite boundaries form in approximately the middle of the dorsal metameric units (red line); consequently the tergites are shifted compared to the ventral segmental units in basal non-ring-forming millipedes like *Glomeris*. (B) The model can be extrapolated to ring-forming millipedes as indicated by blue shading. In most ring-forming millipedes tergite II will fuse with ventral segment 1, tergite III with ventral segment 2, and so on. (C) However, in some ring-forming millipedes (Spirobolida) tergite II will fuse with ventral segment 2, tergite III with ventral segment 3, and so on. For details, see text.

In *Glomeris*, the first eight pairs of legs as well as the first six tergites form before the animal hatches. Three different kinds of boundaries can be distinguished that subdivide the trunk of the *Glomeris *embryo into three distinct series of metameric units. On the ventral side, metameric boundaries delimit a series of eight metameric units. These units correspond to the number of trunk sternites and leg pairs that are specified during embryogenesis. We have referred to these segmental units as ventral embryonic segments. On the dorsal side, there are two different kinds of boundaries: first, there are the boundaries that subdivide the dorsal epidermis into a series of six metameric units, which are the lateral plates of Dohle [4]. We designated these dorsal metameric units as dorsal embryonic segments. Second, there are the boundaries between the tergites that subdivide the dorsal trunk epidermis into a series of six metameric units that correspond to the number of tergites that are specified during embryogenesis. These boundaries form later in embryogenesis than the boundaries between the dorsal and ventral embryonic segments. They form approximately in the middle of the dorsal embryonic segments coinciding with the dorsal stripes of *engrailed *expression. Thus, the tergites in *Glomeris *consistently span from the middle of one dorsal embryonic segment to the middle of the next dorsal embryonic segment (Fig. 9A). This finding is in contrast to earlier beliefs that the borders between the tergites must align with other metameric boundaries in the trunk (see previous models). The displacement of the tergites in relation to the other metameric body units is the source of the hitherto contradicting models of tergite/sternite correlation in diplopods.

Our data show that the tergite borders are defined independently from the embryonic segment borders (both ventral and dorsal) and are established by a distinct genetic mechanism that uses *engrailed *but not *wingless *signalling. Thus, in terms of genetic mechanisms involved, the segmentation of the dorsal exoskeleton appears to be different from the segmentation processes leading to the formation of the ventral and dorsal embryonic segments. On the other hand, the formation of the tergites seems to build on the prior establishment of the dorsal embryonic segments. Tergite borders invaginate roughly in the middle of each dorsal embryonic segment and the tergites themselves are formed by the posterior half of one dorsal embryonic segment and the anterior half of the following one. This process bears some resemblance to resegmentation in vertebrates where the primary metameric units, the somites, are divided in the middle and the final metameric units, the vertebral bones, are formed from the posterior half of one somite and the anterior half of the next one (reviewed in [15]). The two processes in *Glomeris *and vertebrates certainly are not homologous, but might nevertheless share some developmental mechanisms by convergent evolution. These new insights now allow a modified model for tergite – sternite/leg pair correlations in basal milipedes with non-fused exoskeletal elements, like *Glomeris *(see Fig. 9A). But our data also have ramifications to the ring-forming process in derived millipedes. According to our results, the tergites are shifted in relation to the sternites on the ventral side of the animals. In order to fuse into a ring, a tergite therefore has to "choose" between two alternatives: for example tergite III may fuse either with the sternite a bit in front of it (sternite of ventral embryonic trunk segment 2), or with the sternite a bit behind of it (sternite of ventral embryonic trunk segment 3). The former alternative where the tergites fuse with the sternites shifted slightly in front of them would lead to the situation found in the large majority of all ring-forming species (Fig. 9B): tergites II, III, and IV fuse with sternites 1, 2, and 3 respectively, whereas all following tergites fuse with two consecutive sternites, leading to three rings with a single leg pair followed by rings with two leg pairs each. However, the second alternative also seems to be realized in nature, because there is a group of juliform millipedes, the Spirobolida, that have four single leg bearing rings [16]. Assuming that in these animals the tergites fuse with the sternites of the slightly posterior ventral embryonic segments, the result would be four rings with a single leg pair followed by rings with two leg pairs each (Fig. 9C).

Although our results suggest a solution to the problem of tergite – sternite/leg pair correlations in diplopods, they do not provide an immediate answer as to the origin and evolution of diplosegments in this myriapod group. Our results do not support the existence of single segments with two pairs of legs each. Rather, the expression of *engrailed*, *wingless *and other segmentation genes [6] suggests that the ventral embryonic segments correspond to the body segments of other arthropods and these ventral embryonic segments bear only a single pair of legs each. The term "segment" is problematic when used for metameric units in millipedes, because as we have shown during embryogenesis there are at least three different series of repeated units (ventral embryonic segments, dorsal embryonic segments, tergites) all of which are established independent from each other. In the past the term "segment" has been used quite indiscriminately for all these repeated units and also for composite units like the fused armour rings in ring-forming species (for an extended discussion of arthropod segments and their homology, see Ref. [17] and [18]). Our data reject the idea that the rings in adult ring-forming species are homologous to the body segments of other arthropods. Rather, these rings seem to be a specific innovation of these diplopod species and are a composite of parts from different metameric units. In order to avoid further confusion in the future, we suggest not to use the term "segment" for the body rings and to denote the rings with a single leg pair as "haplorings" instead of "haplosegments" and the rings with two pairs of legs as "diplorings" instead of "diplosegments".

It is clear now, that the origin of diplosegmentation cannot be understood on the basis of the tergites alone, but must also take into account the origin and development of the dorsal embryonic segments, especially those that correlate with two ventral embryonic segments. Unfortunately, so far we were not able to identify genes that are expressed at the boundaries between the dorsal embryonic segments; thus at present the establishment of this boundary is unclear. Research into the developmental genetics of the dorsal embryonic segments in *Glomeris *is thus an exciting topic and will lead to further insights into the peculiar mode of diplosegmentation in the future.

Apart from the still unclear mode of diplosegmentation, however, our finding of the independent segmentation of the dorsal exoskeleton (tergites) in *Glomeris*, may explain dorsoventral peculiarities in other arthropods. As mentioned in the introduction, taxa like *e.g. *notostracan crustaceans, pauropods and symphylans show a mismatch of tergite numbers with leg pairs. Independent dorsal and ventral segmentation in a similar manner as in *Glomeris*, and the independent establishment of the tergites, in theory can lead to any combination of tergites with any number of leg pairs. If tergite formation and the formation of segments also are not coupled in notostracans, pauropods, and symphylans, this could be the cause for the dorsoventral discrepancies in these arthropods.

## References

[B1] Enghoff H, Dohle W, Blower JG (1993). Anamorphosis in millipedes (Diplopoda) – the present state of knowledge with some developmental and phylogenetic considerations. Zool J Linn Soc.

[B2] Wilson HM (2002). Muscular anatomy of the millipede *Phyllogonostreptus nigrolabiatus *(Diplopoda: Spirostreptida). J Morph.

[B3] Heymons R (1897). Mittheilungen über die Segmentirung und den Körperbau der Myriopoden. Sitzungsberichte der preussischen Akademie der Wissenschaften zu Berlin.

[B4] Dohle W (1964). Die Embryonalentwicklung von *Glomeris marginata *(Villers) im Vergleich zur Entwicklung anderer Diplopoden. Zool Jb Anat.

[B5] Dohle W (1974). The segmentation of the germ band of Diplopoda compared with other classes of arthropods. Symp Zool Soc London.

[B6] Janssen R, Prpic NM, Damen WGM (2004). Gene expression suggests decoupled dorsal and ventral segmentation in the millipede *Glomeris marginata *(Myriapoda: Diplopoda). Dev Biol.

[B7] Nüsslein-Volhard C, Wieschaus E (1980). Mutations affecting segment number and polarity in *Drosophila*. Nature.

[B8] Lawrence PA (1988). The present status of the parasegment. Development.

[B9] Lawrence PA (1992). The Making of a Fly: The Genetics of Animal Design.

[B10] Lawrence PA, Johnston P, Macdonald P, Struhl G (1987). Borders of parasegments in *Drosophila *embryos are delimited by the *fushi tarazu *and *even-skipped *genes. Nature.

[B11] Martinez-Arias A, Lawrence PA (1985). Parasegments and compartments in the *Drosophila *embryo. Nature.

[B12] Damen WGM (2002). Parasegmental organization of the spider embryo implies that the parasegment is an evolutionary conserved entity in arthropod embryogenesis. Development.

[B13] Hughes CL, Kaufman TC (2002). Exploring myriapod segmentation: the expression patterns of *even-skipped, engrailed*, and *wingless *in a centipede. Dev Biol.

[B14] Scholtz G, Patel NH, Dohle W (1994). Serially homologous *engrailed *stripes are generated via different cell lineages in the germ band of amphipod crustaceans (Malacostraca, Peracarida). Int J Dev Biol.

[B15] Saga Y, Takeda H (2001). The making of the somite: molecular events in vertebrate segmentation. Nature Reviews Genetics.

[B16] Bodine MW (1970). The segmental origin of the appendages of the head and anterior body segments of a spiroboloid milliped, *Narceus annularis.*. J Morph.

[B17] Budd GE (2001). Why are arthropods segmented?. Evol Dev.

[B18] Minelli A, Fusco G Evo-devo perspectives on segmentation: model organisms, and beyond. Trends in Ecology and Evolution.

[B19] Dohle W, Westheide W, Rieger R (1996). Progoneata. Spezielle Zoologie, Teil 1: Einzeller und Wirbellose Tiere.

